# Postoperative Pain Following Eye Enucleation: A Prospective Observational Study

**DOI:** 10.3390/medicina60040614

**Published:** 2024-04-09

**Authors:** Nicolas Leister, Johannes Löser, Antoniu-Oreste Gostian, Magdalena Gostian, Alexander C. Rokohl, Marc A. Fieber, Deniz Alkan, Christine Schumacher, Vanessa Löw, Erik Gordon, Bernd W. Böttiger, Ludwig M. Heindl

**Affiliations:** 1 Department of Anesthesiology and Intensive Care Medicine, Faculty of Medicine and University Hospital Cologne, University of Cologne, 50937 Cologne, Germany; loeser.johannes@googlemail.com (J.L.); marc.fieber@uk-koeln.de (M.A.F.); deniz.alkan@uk-koeln.de (D.A.); christine.schumacher@uk-koeln.de (C.S.); vanessa.loew@uk-koeln.de (V.L.); bernd.boettiger@uk-koeln.de (B.W.B.); 2 Department of Palliative Care Medicine, Faculty of Medicine and University Hospital Cologne, University of Cologne, 50937 Cologne, Germany; 3 Department of Otolaryngology, Head & Neck Surgery, University Hospital Erlangen, Friedrich-Alexander-University of Erlangen-Nuremberg, 91054 Erlangen, Germany; antoniu-oreste.gostian@uk-erlangen.de; 4 Department of Anesthesiology and Intensive Care Medicine, Malteser Waldkrankenhaus St. Marien, 91054 Erlangen, Germany; magdalenaf@gmx.de; 5 Department of Ophthalmology, Faculty of Medicine and University Hospital Cologne, University of Cologne, 50937 Cologne, Germany; alexander.rokohl@uk-koeln.de (A.C.R.); egordon.stud@gmail.com (E.G.); ludwig.heindl@uk-koeln.de (L.M.H.)

**Keywords:** anophthalmic surgery, pain assessment, pain therapy, therapy side-effects

## Abstract

*Background and Objectives:* Enucleation of an eye is the most invasive procedure in ophthalmologic surgery. It can be the result of various diseases (malignant/chronic/trauma/infection) and is nevertheless relatively rare, but leads to the loss of a strongly innervated neuronal organ. This study systematically evaluates postoperative pain levels following enucleation of the eye globe. *Materials and Methods:* This prospective single-center study enrolled twenty-four patients undergoing enucleation of the eye globe. Perioperatively all patients completed (preoperative day, day of surgery, 1st, 2nd, and 3rd day following surgery) standardized questionnaires concerning their pain experience and treatment-related side-effects (internal protocol, QUIPS, painDETECT^®^). Patients received usual pain therapy in an unstandardized individual manner. *Results:* Preoperatively, mean average pain intensity of all included patients was 3.29 ± 2.46 (range, 0–8), 3.29 ± 3.24 (range, 0–8) on the day of surgery, 4.67 ± 1.90 (range, 2–10) on day 1, 3.25 ± 1.39 (range, 1–6) on day 2, and 2.71 ± 1.30 (range, 1–6) on day 3 after surgery. Mean maximum pain intensity was 4.71 ± 3.28 (range, 0–10) preoperatively, 4.04 ± 3.78 (range, 0–10) on the day of surgery, 5.75 ± 2.01 (range, 2–10) on day 1, 4.25 ± 1.89 (range, 2–10) on day 2, and 3.88 ± 1.54 (range, 2–8) on day 3 after surgery. Nineteen patients (79.2%) stated that they would have preferred more pain therapy. *Conclusions:* Patients undergoing eye enucleation report pain sensations in need of intervention in this university hospital. Thus, effective standardized pain treatment concepts are now a high priority to be established in an interdisciplinary manner containing standardized regimens and continuous regional procedures. Awareness of this problem in the medical team should be sharpened through targeted training and information.

## What Is Already Known on This Topic

Enucleation of the eye globe is an incisive and one of the most invasive ophthalmic surgical procedures. Little is known about the pain experienced by patients pre-, peri-, and postoperatively or the best pain management for enucleation.

## What This Study Adds

Patients undergoing eye globe enucleation suffer pain in need of intervention pre-, peri-, and postoperatively when pain is treated in an unstandardized manner at the discretion of the attending physician.

## How This Study Might Affect Research, Practice, or Policy

Awareness of the pain problem among the medical team should be raised through targeted training and information. Pain treatment concepts should be developed that include standardized regimens and possibly continuous regional procedures. Further studies are needed to reduce the pain burden on patients.

## 1. Introduction

Removal of the eye is a rare therapeutic treatment used for end-stage ophthalmic diseases that do not respond to less conservative treatments. Common indications for anophthalmic surgery are intraocular tumors, trauma, infection, secondary glaucoma, and phthisis bulbi [1,2,3]. Indications for removal of the eye are similar in most hospitals and countries. However, the frequency of pathologic conditions leading to this invasive procedure varies by hospital and location [1,2,3]. In European countries, the indication for removal of the eye is usually a malignant condition when other therapeutic options have failed or are not feasible [4,5]. The leading cause for anophthalmic surgery in developing countries is infection or perforating trauma (e.g., gun shot) [1,2,6]. One possible type of anophthalmic surgery is enucleation, which, like the other anophthalmic therapies (e.g., evisceration), is an incisive and invasive surgical ophthalmologic procedure: the entire globe of the affected eye is removed [7,8,9]. After this surgical intervention, which has a drastic effect on the lives of the patients, postoperative pain leads to an acute reduction in well-being and increases the risk of the development of chronic pain [7]. This postoperative chronic pain is called persistent postsurgical pain (PPSP). It lasts more than 3 months after surgery and affects the associated region of innervation in the operated area [10]. This PPSP is one of the most common complications following surgery (around 40%), with half of these patients describing at least mild to severe PPSP [11]. Many factors influence the development of PPSP, although some (e.g., psychological factors) cannot necessarily be directly influenced by the surgeon. Others (e.g., surgical technique, intraoperative nerve injury, postoperative pain management), however, can be directly modified by the medical team. For this reason, adequate acute pain therapy is essential to prevent the chronification of postoperative pain [12]. In the special case of eye globe removal, there is an association with an increase in the risk of suffering phantom eye pain, when severe postoperative pain is experienced [13,14]. In many ophthalmic surgical procedures, pain is not a major concern (e.g., Descemet membrane endothelial keratoplasty) or has not been well addressed [15]. Considering the importance of sufficient and effective pain management for patients’ well-being both in the short term and later in life, we wanted to identify what degree of pain patients undergoing enucleation of the eye experience in our center and whether the usual, non-standardized individual pain management is sufficiently effective [16,17]. Therefore, pain sensation levels (primary objectives), efficacy of individual pain management at the discretion of the treating physician, and treatment-related side effects (secondary objectives) were recorded and evaluated in the present study: All included patients were interviewed once a day using an in-house hospital pain questionnaire on the preoperative day, on the day of surgery, and on each of the first three postoperative days (POD). Patients were asked about average and maximum pain using the numeric rating scale (NRS). Furthermore, patients completed the Quality Improvement in Postoperative Pain Management (QUIPS) questionnaire on the first POD [18] and the painDETECT^®^ questionnaire for neuropathic pain on the third POD [19,20].

## 2. Materials and Methods

### 2.1. Ethics Committee Approval

This study was approved by the responsible ethics committee (No: 17-247; 1 July 2019) of the University Hospital of Cologne, Cologne, Germany. The study was registered in the German Clinical Trials Register on 8 February 2018 (DRKS00013995). Informed consent was obtained from all subjects and/or their legal guardian(s). The study was conducted in accordance with the Helsinki Declaration on Patient Safety [21].

### 2.2. Patient and Data Selection—Inclusion Criteria

Patients who underwent enucleation at the Department of Ophthalmology at the University Hospital of Cologne, Germany from March 2018 to March 2022 were consecutively enrolled in this prospective study.

Inclusion criteria were:

- Informed patient consent
- Body weight between 50 and 120 kg
- Age over 18 years
- American Society of Anesthesiologists (ASA) score 1 to 3.

Exclusion criteria were: Patients with massive language or comprehension problems. Furthermore, patients with intolerance to analgesic drugs (ibuprofen, metamizole, tilidine, oxycodone, or morphine) were also excluded.

Pain management during the perioperative hospitalization was performed at the discretion of the attending physician. There was no standardized pain regimen in this university hospital before and during the study period. Therefore, all patients received non-standardized, individualized pain therapy.

### 2.3. Anesthesia and Recovery Room

In all patients, general anesthesia was induced according to the current hospital standard (propofol, remifentanil, atracurium) intravenously. To maintain anesthesia, balanced anesthesia (desflurane or sevoflurane, remifentanil) was used. During ongoing surgery, patients received piritramide (0.05–0.2 mg/kg bodyweight) to treat postoperative pain. Pain in the recovery room was treated with another bolus of piritramide (0.05–0.2 mg/kg bodyweight) up to a pain intensity of <3 (numeric rating scale).

### 2.4. Surgery

Following retrobulbar anesthesia (lidocaine and buprenorphine with adrenaline), the entire eye globe was removed by severing the attachments of the conjunctiva at the limbus, the extraocular muscles, and the optic nerve. An orbital implant (coralline hydroxyapatite coated with a vicryl mesh) of adequate volume was inserted within Tenon’s capsule. A conformer was embedded in the sutured conjunctiva, until an ocular prosthesis was manufactured (usually 10 to 14 days after surgery) [9,22].

### 2.5. Detection of Patients’ Pain Intensity and Well-Being

Patients were interviewed once a day using an in-house hospital pain questionnaire on the preoperative day, on the day of surgery, and on each of the first three POD. Patients reported average and maximum pain using NRS. Furthermore, patients were asked about treatment-associated side effects. In addition, participating patients completed the Quality Improvement in Postoperative Pain Management (QUIPS) questionnaire on the first POD [16,17]. Furthermore, on the third POD, patients completed the painDETECT^®^ questionnaire for neuropathic pain [19,20].

### 2.6. In-House Daily Pain Questionnaire

This questionnaire records how often patients have pain and how severe the pain is: NRS from 0 (no pain) to 10 (strongest imaginable pain). The average pain intensity and the strongest pain felt were asked using the NRS. Non-drug measures for pain, the use of medication (painkillers), and possible treatment-related side effects (e.g., sleep disorders, dizziness, nausea, and impaired concentration) were also recorded. Patients’ opinions were recorded on whether they would have preferred more pain medication.

### 2.7. QUIPS Questionnaire

QUIPS is an interdisciplinary and multicenter benchmark project that aims to improve acute, postoperative pain management in surgical centers (standardized quality indicators are collected and evaluated). The questions asked in this questionnaire were both yes/no as well as questions that were rated on a scale from 0 (lowest value) to 15 (highest value) [17,18].

### 2.8. painDETECT^®^ Questionnaire

The patient is asked about the intensity of pain (current, average of the last 4 weeks, and most severe pain of the last 4 weeks), localization, characteristics, quality, and symptoms. In this way, the painDETECT^®^ questionnaire is used to identify a neuropathic pain sensation with numerical values. The higher the score, the higher the likelihood of the presence of a neuropathic pain component. Scores above 18 indicate a high probability of a neuropathic pain component. Scores below 13 make a neuropathic pain component very unlikely [19,20].

### 2.9. Statistical Analysis

The statistical analysis was carried out using SPSS version 27.0 (IBM, SPSS Statistics, IBM Corporation, Chicago, IL, USA).

Distribution of demographic data (age, etc.) is presented as mean (range). Further data are given as mean +/− standard deviation (range).

ANOVA (and post hoc) analyses were performed to determine significant changes in pain perception between days. All *p* values below 0.05 were considered statistically significant.

## 3. Results

### 3.1. Demographic Data

Between February 2018 and December 2021, a total of 24 patients (11 men/13 women) underwent enucleation of the eye in general anesthesia in combination with retrobulbar blockade (lidocaine and buprenorphine with adrenaline). The mean age was 66 years (range, 27–85 years). Indications for eye enucleation are listed in Table 1.

### 3.2. Average Pain

**Figure 1:**
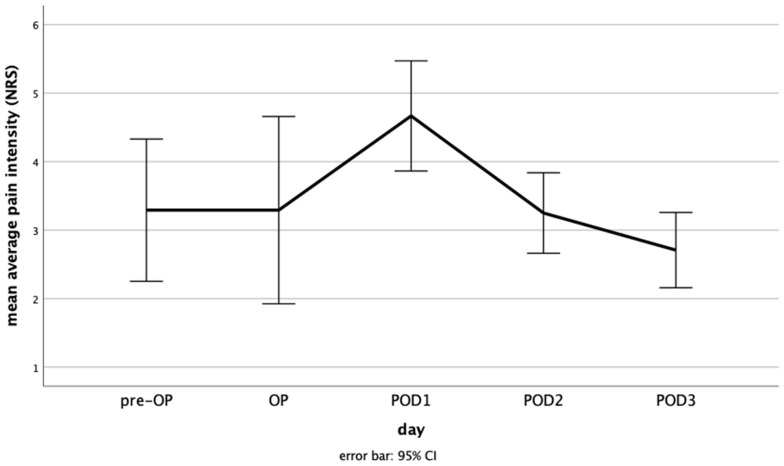
Mean average pain intensity from the preoperative day to the third postoperative day. NRS, numeric rating scale; pre-OP, preoperative day; OP, day of surgery; POD, postoperative day; CI, confidence interval. Mean average pain intensity on the day of surgery was as high as on the preoperative day, there was a maximum on POD1, and mean average pain intensity decreases on POD3 to values below the preoperative and day of surgery levels.

Preoperatively, mean average pain intensity of the patients was 3.29 ± 2.46 (range, 0–8). On the day of surgery, the mean average pain level was 3.29 ± 3.24 (range, 0–8). It was 4.67 ± 1.90 (range, 2–10) on POD1, 3.25 ± 1.39 (range, 1–6) on POD2, and 2.71 ± 1.30 (range, 1–6) on POD3 (Figure 1). ANOVA showed significant differences between the days with *p* = 0.035. The post hoc test showed significance for mean average pain intensity on POD1 compared to all other days (preoperative day *p* = 0.031; day of surgery *p* = 0.031; POD2 *p* = 0.026; POD3 *p* = 0.002).

### 3.3. Maximum Pain

**Figure 2:**
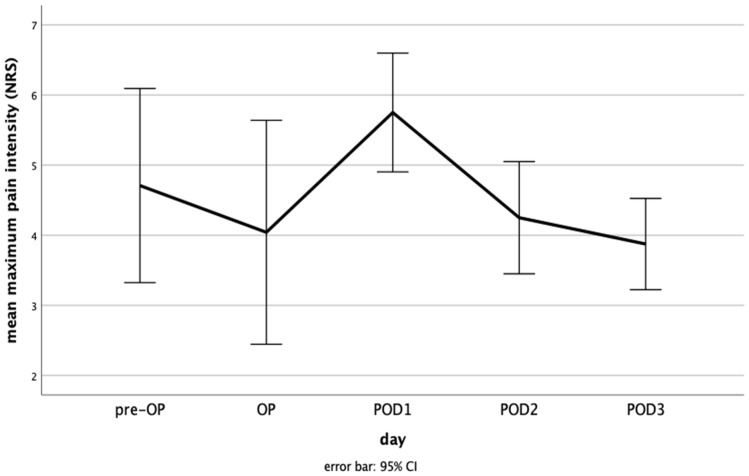
Mean maximum pain intensity from the preoperative day to the third postoperative day. NRS, numeric rating scale; pre-OP, preoperative day; OP, day of surgery; POD, postoperative day; CI, confidence interval. Mean maximum pain intensity was already relatively high on the preoperative day, there was a maximum on POD1, and mean maximum pain intensity decreases on POD2 and POD3 to values below the preoperative levels.

Preoperatively, the mean maximum pain intensity was 4.71 ± 3.28 (range, 0–10). On the day of surgery, mean maximum pain intensity was 4.04 ± 3.78 (range, 0–10). On POD1 it was 5.75 ± 2.01 (range, 2–10), 4.25 ± 1.89 (range, 2–10) on POD2, and 3.88 ± 1.54 (range, 2–8) on POD3 (Figure 2). ANOVA showed no significant differences between the days with *p* = 0.108. The post hoc test showed significance for mean maximum pain intensity on POD1 compared to day of surgery (*p* = 0.27) and to POD3 (*p* = 0.016).

### 3.4. QUIPS Questionnaire

At the time of the survey, only one patient felt no pain at all. No patients expressed pain in the recovery room, but 23 patients did on the normal ward.

### 3.5. PainDETECT^®^ Questionnaire

In 12 patients (50%), the PainDETECT^®^ questionnaire showed a negative result (<13) for neuropathic pain. None of the patients showed a positive result (>18) for neuropathic pain, but 12 patients (50%) had a value between 13 and 18, thus an unclear result.

### 3.6. Perioperative Consumption of Analgesics

Preoperatively, 54.17% of the participants (13/24) used analgesics (non-opioids and opioids). All of the patients received analgesics on day of surgery. On POD1, 87.5% (21/24), on POD2, 66.67% (16/24), and on POD3, 37.5% of patients used analgesics (non-opioids and opioids).

### 3.7. Desire for Further Pain Therapy

Nineteen patients (79.2%) stated that they would have preferred more pain therapy than they received.

### 3.8. Treatment-Related Side-Effects

#### 3.8.1. Nausea

The mean nausea score on the day before surgery was 0.33 ± 1.63 (range, 0–8), with only one patient suffering from nausea. On the day of surgery, the mean nausea score was 1.13 ± 2.40 (range, 0–8), 0.71 ± 2.25 (range, 0–10) on POD1, 0.17 ± 0.64 (range, 0–3) on POD2, and 0.04 ± 0.2 (range, 0–1) on POD3.

#### 3.8.2. Emesis

Preoperatively, no patient mentioned emesis. On the day of surgery, the mean emesis score was 0.33 ± 1.43 (range, 0–7), and on POD2 the mean emesis score was 0.17 ± 0.64 (range, 0–3). On POD1 and POD3, no patient reported emesis.

#### 3.8.3. Constipation

Preoperatively, mean constipation score was 0.29 ± 1.08 (range, 0–5). On the day of surgery, the reported constipation score was 0.33 ± 1.63 (range, 0–8), 0.96 ± 2.33 (range, 0–10) on POD1, 0.83 ± 2.41 (range, 0–10) on POD2, and 0.54 ± 1.89 (range, 0–8) on POD3.

#### 3.8.4. Fatigue

Preoperatively, mean fatigue score was 0.54 ± 1.32 (range, 0–5). On the day of surgery, mean fatigue score was 2.54 ± 2.60 (range, 0–8), 2.42 ± 3.26 (range, 0–10) on POD1, 1.62 ± 2.72 (range, 0–10) POD2, and 0.96 ± 1.92 (range, 0–7) on POD3.

#### 3.8.5. Concentration Disorders

Preoperatively, a mean concentration disorder score of 1.54 ± 2.47 (range, 0–9) was reported. On the day of surgery, the mean concentration disorder score was 1.21 ± 2.30 (range, 0–8), 1.92 ± 3.35 (range, 0–10) on POD1, 1.13 ± 2.92 (range, 0–10) on POD2, and 0.63 ± 1.74 (range, 0–7) on POD3.

#### 3.8.6. Sleep Disorders

Preoperatively, an average sleep disorder score of 0.96 ± 2.05 (range, 0–7) was reported. On the day of surgery, the mean sleep disorder score was 1.33 ± 2.85 (range, 0–10), 2.42 ± 3.49 (range, 0–10) on POD1, 1.54 ± 2.77 (range, 0–10) on POD2, and 0.63 ± 1.74 (range, 0–7) on POD3.

#### 3.8.7. Vertigo

Preoperatively, a mean vertigo score of 0.42 ± 1.41 (range, 0–5) was reported. On the day of surgery, the mean vertigo score was 0.04 ± 0.21 (range, 0–1), 1.17 ± 2.68 (range, 0–10) on POD1, and 0.38 ± 1.13 (range, 0–5) on POD2. On POD3, no patient reported vertigo.

## 4. Discussion

Surgical procedures in the ophthalmic area are frequently considered to be less painful, which leads to the fact that pain therapy in ophthalmology tends to be largely underestimated [11,15,23,24]. However, the enucleation of an eye is a drastic intervention that leaves visual and psychological traces on the patient [4,7,8]. Often, these patients have already suffered from a long history of pain, which leads to the final decision to have the eye removed. The current and earlier literature indicates that preoperative pain predisposes to a complicative postoperative pain experience. Moreover, personal characteristics such as distinct pain catastrophizing, anxiety, and habits such as smoking or an elevated body mass index (BMI) can lead to complex pain scenarios and increased incidence of chronic pain [25,26,27,28,29]. In particular, smoking and preoperative anxiety were found to be independent risk factors for postoperative pain in anophthalmic surgery [30]. Furthermore, a comprehensive, individual pre-anesthesiology visit can reduce the patient’s anxiety about the uncertainties, and thus probably also the risk of pain chronification [31]. However, these factors (anxiety, pain catastrophizing, BMI, smoking) were not included in the study design.

Previous studies have shown that sufficient pain therapy can counteract a subsequent phantom pain [13,14]. Due to this circumstance and the fact that pain also disturbs the current well-being of patients, sufficient and effective pain therapy is mandatory. However, the status quo in modern ophthalmic surgery is usually poorly known. With this in mind, the present study is intended to narrow the knowledge gap to some degree by investigation of preoperative pain scores and pain up to three days after surgery in patients undergoing eye enucleation.

In the present study, it is shown that patients undergoing enucleation of an eye suffer from relevant pain in this university hospital. It is obvious that the patients already report average pain levels in need of intervention preoperatively (mean average 3.29); also, with regard to the pain peaks, a remarkably high value of 4.71 is obtained. This indeed raises the question whether a more intensive pain therapy should be initiated preoperatively in spite of or just because of terminal eye disease. Thus, in the German guidelines, an intervention limit would be defined as NRS > 3 or if the patient wishes for an improvement of analgesic administration [32]. Furthermore, the data suggest that pain therapy (with intraoperative retrobulbar block and systemic opioid therapy) is still very sufficient in the recovery room immediately after surgery and that the patients are in little pain, although a fairly high level of pain is already reached on the normal ward during the course of the day of surgery (mean average 3.29; mean maximum 4.04.), which rises to a maximum on the first postoperative day (mean average 4.67; mean maximum 5.75). This indicates that an individual pain therapy without a mandatory regimen does not seem to be sufficient for these patients suffering pain in this university hospital. For this reason, a sufficient pain assessment and prophylactic therapy in the sense of an interdisciplinary pain therapy concept seems to be imperative, starting preoperatively. In addition, continuous regional anesthesia procedures (e.g., catheter-based) should be discussed in order to reduce the patients’ pain perception at least in the first two postoperative days; under certain circumstances, the rate of phantom pain could also be influenced in this way.

The fact that 79.2% of the patients wished for additional or improved pain therapy is indeed worth considering: Patients expect to be optimally treated with pain therapy methods despite, or especially, when undergoing highly invasive procedures. This requires the entire medical team to respond to expectations not simply with pharmaceuticals but also with non-pharmacological techniques (such as distraction, physiotherapy, etc.). However, it is also crucial that it is conveyed from the very beginning that complete relief from pain may not be possible. This can help to temper unrealistic expectations. The interdisciplinary approach to perioperative pain and the fact that the patient could influence pain sensations by performing appropriate procedures must be communicated. Complaints regarding pain must always be taken seriously, as it is a subjective perception. The team providing care must be alert to this issue. However, this should lead to a standardization of pain therapy with individual adaptation as well as an increase in awareness of this problem among the attending medical staff (doctors and nursing staff).

With regard to the treatment-related side effects, a maximum can be observed on the first postoperative day in all the categories considered. Taking this aspect into account, the high pain values on the first postoperative day and the patients’ pronounced desire for additional pain therapy could lead to the conclusion that an under-therapy or insufficient therapy of the pain is causing these effects.

The incidence of postoperative sleep disorders appears to be low, which is surprising given the loss of retinal ganglion cells, which are responsible for the circadian machinery [33,34,35]. The literature to date indicates that enucleation of the eye may facilitate the pathway to chronic sleep disorders [36]. Unfortunately, however, the types of sleep disorder (nightmares, insomnia, etc.) have not been recorded in this study.

The results of the present study indicate that patients undergoing eye enucleation suffer pain in need of intervention in this university hospital when pain therapy is performed in this usual unstandardized individual manner provided at the discretion of the attending physician. Effective pain therapy concepts should be developed in an interdisciplinary manner and contain standardized regimens and possibly continuous regional procedures [37,38,39]. Awareness of this problem in the medical team should be sharpened through targeted training and information.

### Limitations

The limited number of patients included is due to the research topic. The sample size may therefore affect the statistical power of the study, and evidence of effectiveness or ineffectiveness of pain treatment strategies or the assessment of pain levels may not be as robust as in a wider patient population. Moreover, individual factors such as anxiety, pain catastrophizing, BMI, or smoking were not recorded by the study design. Therefore, data on these individual factors influencing the perception of pain are not available. Being a single-center study, it has all the limitations going along with these studies.

In the current study, there is furthermore a lack of a proper control group. As a result, the study is not able to show pain therapy improvements directly attributable to interventions as opposed to the usual recovery process or even placebo effects. Although the patients were informed in advance about the standardized procedure of the study, a bias cannot be ruled out through direct contact with the examiner and the presence of a bedfellow may also have influenced the answers. Some patients were operated in the morning and others in the afternoon, and the influence of this cannot be ruled out since the interview took place in the evening.

Future prospective trials will have to analyze the effect of standardized analgesia concepts and the potential effect of the development of continuous catheter-based regional anesthesia procedures.

## 5. Conclusions

In summary, patients undergoing eye globe enucleation suffer pain in need of intervention when pain is treated in an unstandardized manner at the discretion of the attending physician in this university hospital. Pain treatment concepts should be developed in an interdisciplinary manner, and contain standardized regimens and possibly continuous regional procedures. Awareness of this problem in the medical team should be sharpened through targeted training and information.

## Figures and Tables

**Table 1:** Indications for eye enucleation.

Indication	Number
Bacterial infection	1
Ciliary body tumor	1
Conjunctival tumor	1
Autoimmune inflammation	2
Perforating trauma	3
Glaucoma (phthisis bulbi)	6
Choroidal melanoma	10

## Data Availability

All data are available upon reasonable request at the corresponding author.
